# Slow-Release Oral Morphine vs Methadone for Opioid Use Disorder in the Fentanyl Era

**DOI:** 10.1001/jamanetworkopen.2026.2970

**Published:** 2026-03-24

**Authors:** M. Eugenia Socias, Jingxin Lei, Vivienne Zhou, Rohan Anand, Zishan Cui, Stephanie Penta, Marc Larochelle, Sara Lodi, Nadia Fairbairn

**Affiliations:** 1 British Columbia Centre on Substance Use, Vancouver, British Columbia, Canada; 2 Division of Social Medicine, Department of Medicine, University of British Columbia, Vancouver, British Columbia, Canada; 3 Section of General Internal Medicine, Department of Medicine, Boston Medical Center and Boston University School of Medicine, Boston, Massachusetts; 4 Department of Biostatistics, Boston University School of Public Health, Boston, Massachusetts

## Abstract

**Importance:**

Slow-release oral morphine (SROM) is approved for the treatment of opioid use disorder (OUD) in several European countries and has been used off label in Canada since 2017. However, the evidence base to support its use is limited and predates the fentanyl era.

**Objective:**

To compare the discontinuation rates and mortality risks of SROM vs methadone for the treatment of OUD.

**Design, Setting, and Participants:**

This comparative effectiveness study with a target trial emulation approach used data from observational linked health administrative databases in the Vancouver Coastal Health Authority (VCHA) region in British Columbia, Canada, between July 1, 2017, and June 30, 2024. Participants were adults aged 19 to 65 years with OUD.

**Exposures:**

New prescription of SROM or methadone for OUD (ie, no dispensation of medications for OUD in the 7 days prior) within VCHA clinical services.

**Main Outcomes and Measures:**

Estimated 12-month risk of treatment discontinuation (≥7 days without medication dispensation) and all-cause mortality, as well as treatment adherence (proportion of days covered [PDC]) over 12 months of follow-up. Both intention-to-treat (ITT) and per-protocol (PP; restricted to episodes where SROM or methadone was dispensed within 4 days of the prescription) analyses were conducted.

**Results:**

A total of 3254 unique individuals (median age, 37 [IQR, 30-46] years; 2088 [64.2%] male) contributed to 4059 person-trials (2737 [67.4%] methadone, 1322 [32.6%] SROM) for the ITT analysis, and 1992 unique individuals contributed to 2276 person-trials (1653 [72.6%] methadone, 623 [27.4%] SROM) for the PP analysis. In ITT analyses, the standardized cumulative incidence of discontinuation at 12 months was 99.3% (95% CI, 99.0%-99.5%) and 97.9% (95% CI, 97.5%-98.2%) in the SROM and methadone arms, respectively (adjusted risk difference [ARD], 1.4 percentage points [pp]; 95% CI, 1.0-1.9 pp). PDC was also lower in the SROM group (adjusted risk ratio, 0.89; 95% CI, 0.87-0.91), while mortality risk was similar between arms (ARD, 1.1 pp; 95% CI, −1.3 to 4.1 pp). Of 121 total deaths, only 2 (1.7%) occurred when people were receiving MOUD. PP analyses yielded similar results.

**Conclusions and Relevance:**

In this comparative effectiveness study of adults with OUD in a Canadian setting, treatment discontinuation was common. Compared with methadone, SROM was associated with a slightly higher risk of treatment discontinuation and lower adherence but similar mortality risk. The findings suggest either medication may be appropriate if measures are in place to support retention.

## Key Points

**Question:**

What are the comparative discontinuation rates and mortality risks of slow-release oral morphine (SROM) vs methadone for the treatment of opioid use disorder (OUD) in the fentanyl era?

**Findings:**

In this comparative effectiveness study that included 4059 person-trials in British Columbia, Canada, the rate of OUD treatment discontinuation at 12 months was slightly higher for people prescribed SROM vs methadone (99% vs 98%). The mortality risk was similar, and only 2 deaths occurred when people were receiving treatment.

**Meaning:**

In this study, SROM for OUD was associated with slightly higher treatment discontinuation risk than methadone but similar mortality risk, suggesting either medication may be appropriate alongside measures to support retention.

## Teaser

This comparative effectiveness study examines the discontinuation rates and mortality risk of slow-release oral morphine vs methadone for the treatment of opioid use disorder in Canadian adults during the fentanyl era.

## Introduction

In 2024, there were 7289 (17.7 per 100 000 population) opioid-related deaths in Canada and 55 005 (16.3 per 100 000 population) in the US, with the majority of these deaths involving illicitly manufactured fentanyl.^1,2^ In Canada, the province of British Columbia (BC) has been particularly affected, with an opioid-related death rate of 40.6 per 100 000 population in 2024 and with fentanyl being detected in 84% of these deaths.^1^

Despite the well-established benefits of medications for opioid use disorder (MOUD) for reducing opioid-related morbidity and mortality,^3,4,5,6^ there are significant individual- and structural-level barriers to uptake of and engagement in MOUD that undermine their population-level benefits.^7^ For example, in 2022, only 25% of the estimated 6 million people in the US with an opioid use disorder (OUD) received MOUD,^8^ with similar low uptake for the BC population,^9^ highlighting the need for novel treatment options to help close this treatment gap and improve health and social outcomes.^10,11,12^

Several small studies have explored the potential of slow-release oral morphine (SROM)–based MOUD (generally using the 24-hour formulation). A 2019 systematic review of randomized clinical trials (RCTs) comparing SROM with methadone found no significant differences between treatments in retention and nonmedical opioid use.^13^ However, due to the small number of RCTs (N = 4) and participants (N = 471), as well as the low methodologic quality of the included studies, the quality of this evidence was assessed as being insufficient to draw any conclusions regarding SROM use in clinical practice. In addition, all of these studies were conducted in Europe, and none of them included individuals using illicit fentanyl. Based on the promising but limited evidence to support their use, SROM-based MOUD are currently available in several European countries^14,15^ and have been increasingly used in Canada as off-label MOUD since 2017.^16,17^ To improve the evidence base for SROM as an MOUD, our team in Vancouver initiated an RCT (pRESTO) to evaluate the comparative effectiveness of SROM vs methadone for the treatment of OUD.^18^ Recruitment started in December 2019, but due to COVID-19 pandemic–related challenges, including restrictions for in-person research, the RCT had to be prematurely stopped in July 2020. To address the objectives of the RCT, we conducted a target trial emulation leveraging the study protocol of the trial and a large, retrospective, population-level dataset of adults with OUD in Vancouver, Canada.^19^ In the current study, we examined the comparative effectiveness of SROM vs methadone for OUD treatment, focusing on the emulated trial’s primary outcome (treatment discontinuation) and its 2 secondary outcomes: treatment adherence and all-cause mortality.

## Methods

### Design, Setting, and Data Sources

This comparative effectiveness study used a target trial emulation approach.^20^ The protocol of the original pRESTO RCT (target trial) and the emulated trial have been described before^19^ and are summarized in eTable 1 in Supplement 1. For the emulated trial, we used deidentified, linked electronic medical records (EMRs) and routinely collected health administrative data from the Vancouver Coastal Health Authority (VCHA) region in BC, Canada, between July 1, 2017, and June 30, 2024 (eTable 2 in Supplement 1). The current study was approved by the University of British Columbia–Providence Health Care research ethics board and followed the International Society for Pharmacoeconomics and Outcomes Research (ISPOR) guideline.^21^ Informed consent was waived by the University of British Columbia–Providence Health Care because we used deidentified secondary administrative data.

VCHA is 1 of the 5 publicly funded regional health authorities in BC and among the most affected by the overdose crisis, particularly in Vancouver’s Downtown Eastside neighborhood—Canada’s largest street-based drug scene, characterized by high rates of poverty, substance use, and homelessness.^22^ Similar to other regional health authorities in BC, VCHA has a low-threshold MOUD program, including office-based care, dispensation of MOUD through community-based pharmacies, and full public coverage of medical care and MOUD. Methadone was the only available MOUD between 1996 and 2010, when buprenorphine-naloxone became available. In 2017, the provincial government endorsed the off-label use of SROM and injectable diacetylmorphine or hydromorphone as MOUD.^23^ At VCHA, both methadone and SROM are prescribed by a clinician (physician or nurse practitioner) and dispensed via daily witnessed ingestion at a community pharmacy.

### Eligibility Criteria

As summarized in eTable 1 in Supplement 1, the emulated trial included adults aged 19 to 65 years who met the following criteria: (1) a diagnosis of OUD, defined as having a relevant *International Classification of Diseases, Ninth Revision (ICD-9)* or *International Statistical Classification of Diseases and Related Health Problems, Tenth Revision (ICD-10)* code for opioid dependence or abuse (eTable 4 in Supplement 1) or at least 1 episode of MOUD prescription recorded in the EMR; (2) a new prescription (ie, no dispensation of short-acting MOUD in the past 7 days [accounting for take-home doses] or 42 days [for extended-release buprenorphine]) (eTable 3 in Supplement 1) of the 24-hour formulation of SROM-based (ie, Kadian) or methadone-based MOUD recorded in the EMR of a primary care or community VCHA clinic between July 1, 2017, and June 30, 2023, to allow for at least 365 days of follow-up; (3) no cancer diagnosis in the past year; (4) no evidence of prolonged corrected QT (QTc) interval (defined as QTc >500 milliseconds, using the Fridericia formula) in the past 3 months; and (5) no prescription of more than 1 MOUD on the same day. We included multiple episodes of SROM- or methadone-based MOUD for individuals who met eligibility criteria more than once but included only the first qualifying episode in each year to avoid overlap of the 365-day follow-up period (eg, if for 1 individual the first qualifying episode started on April 4, 2019, we only considered subsequent episodes after April 3, 2020).

### Outcome and Follow-Up

The primary outcome for the pRESTO RCT was illicit opioid use, measured by biweekly urine drug testing. However, given that urine drug tests are done inconsistently in routine clinical care, the primary outcome for the emulated trial was treatment discontinuation, defined as a gap in the assigned MOUD dispensation greater than or equal to 7 days, in alignment with quality measures on MOUD continuity^24^ and BC clinical practice guidelines (eMethods 1 in Supplement 1 provides details on MOUD episode construction).^25^ Secondary outcomes were treatment adherence, defined as the proportion of days covered (PDC) with the assigned MOUD in the 365-day follow-up period (as a complementary measure of treatment engagement to account for the chronicity of OUD and common cycling in and out of treatment),^26^ and all-cause mortality. The follow-up period started on the day of SROM or methadone prescription. For the discontinuation outcome, follow-up ended at the date of discontinuation, censored at the time of death or at 365 days, whichever occurred first. For the mortality outcome, follow-up continued until death or was censored at 365 days. For adherence (PDC), the observation window (denominator) was defined as the days from prescription until death or 365 days, whichever was earlier.

### Treatment Strategies and Assignment

We compared 2 treatment strategies: a new prescription for SROM-based or methadone-based MOUD, as recorded in individuals’ EMR at baseline. We estimated the observational analogs of the intention-to-treat (ITT) and per-protocol (PP) effects. The ITT analysis estimated the outcome of being prescribed SROM vs methadone at baseline, regardless of whether the prescription was filled. The PP analysis estimated the outcome of being prescribed SROM vs methadone at baseline and having the prescription filled within 4 days of the prescription as per pharmacy records. The 4-day grace period was chosen to align with BC pharmacies’ policy of canceling an MOUD prescription if it is not picked up within this time frame.

### Potential Confounders

We adjusted our analyses for a comprehensive list of patient and prescription characteristics measured during the 365-day period prior to the index date (ie, the date of SROM or methadone prescription). Confounders included sociodemographic variables, medical history, health care utilization, concomitant medications (in the past 90 days), and prescription episode characteristics (more details are provided in Table 1 and eTables 4 and 5 in Supplement 1).

**Table 1.** Baseline Characteristics of Patients Initiating Methadone or SROM in VCHA Clinics, Overall and Stratified by Treatment Group

Variable	All patients (n = 3254)	Person-trials (n = 4059)	Patients by treatment arm		SMD	
			Methadone (n = 2737)	SROM (n = 1322)	Before IP weighting	After IP weighting
**Sociodemographic characteristics**						
Age, median (IQR), y	37 (30-46)	37 (31-46)	37 (31-45)	38 (31-47)	0.111	−0.008
Sex						
Female	1166 (35.8)	1479 (36.4)	993 (36.3)	486 (36.8)	0.010	0.007
Male	2088 (64.2)	2580 (63.6)	1744 (63.7)	836 (63.2)	−0.010	−0.007
Residence in a metropolitan or urban region	3036 (93.3)	3812 (93.9)	2583 (94.4)	1229 (93.0)	−0.055	−0.003
Residence in VCHA	2271 (69.8)	2908 (71.6)	1940 (70.9)	968 (73.2)	0.053	−0.004
Low-income status	574 (17.6)	677 (16.7)	449 (16.4)	228 (17.3)	0.022	−0.015
Homelessness	472 (14.5)	591 (14.6)	394 (14.4)	197 (14.9)	0.014	−0.002
**Medical history**						
Lifetime history of injection drug use	2322 (71.4)	2972 (73.2)	2004 (73.2)	968 (73.2)	0.001	−0.007
Lifetime history of nonfatal overdose	766 (23.5)	962 (23.7)	660 (24.1)	302 (22.8)	−0.030	−0.005
Alcohol use disorder^a^	394 (12.1)	466 (11.5)	310 (11.3)	156 (11.8)	0.015	−0.004
Stimulant use disorder^a^	937 (28.8)	1213 (29.9)	791 (28.9)	422 (31.9)	0.065	−0.001
Sedative drug use disorder^a^	75 (2.3)	95 (2.3)	61 (2.2)	34 (2.6)	0.022	0.011
Less serious mental disorder^a^	874 (26.9)	1071 (26.4)	698 (25.5)	373 (28.2)	0.060	0.004
Serious mental condition^a^	623 (19.2)	798 (19.7)	523 (19.1)	275 (20.8)	0.042	0.002
Chronic pain condition^a^	745 (22.9)	914 (22.5)	578 (21.1)	336 (25.4)	0.099	−0.004
Hepatitis C^a^	116 (3.6)	155 (3.8)	107 (3.9)	48 (3.6)	−0.015	−0.002
Modified Elixhauser Comorbidity Index^a^^,^^b^						
0	2912 (89.5)	3621 (89.2)	2451 (89.5)	1170 (88.5)	−0.033	−0.001
1	238 (7.3)	307 (7.6)	215 (7.9)	92 (7.0)	−0.035	−0.003
≥2	104 (3.2)	131 (3.2)	71 (2.6)	60 (4.5)	0.068	0.004
MOUD use in the past 5 y	2939 (90.3)	3740 (92.1)	2535 (92.6)	1205 (91.2)	−0.052	−0.012
**Health care utilization^a^**						
Attachment to a regular practitioner	715 (22.0)	940 (23.2)	644 (23.5)	296 (22.4)	−0.027	−0.007
ED visits, median (IQR)	2 (0-6)	2 (0-6)	2 (0-5)	2 (1-6)	0.065	0.001
Hospital admissions, median (IQR)	0 (0-1)	0 (0-1)	0 (0-1)	0 (0-1)	0.037	0.004
Received virtual care	126 (3.9)	141 (3.5)	92 (3.4)	49 (3.7)	0.018	0.001
**Concomitant medications^c^**						
Sedative drug dispensation	489 (15.0)	574 (14.1)	353 (12.9)	221 (16.7)	0.102	−0.002
Nonsedative psychiatric drug dispensation	1181 (36.3)	1477 (36.4)	999 (36.5)	478 (36.2)	−0.007	−0.016
Opioid analgesic dispensation	2114 (65.0)	2676 (65.9)	1907 (69.7)	769 (58.2)	−0.233	−0.009
**MOUD episode characteristics**						
Codispensed hydromorphone as safer supply^27^	704 (21.6)	1027 (25.3)	649 (23.7)	378 (28.6)	0.108	0.012
Year of initiation						
2018	546 (16.8)	546 (13.4)	371 (13.6)	175 (13.2)	−0.009	−0.001
2019	801 (24.6)	837 (20.6)	561 (20.5)	276 (20.9)	0.009	−0.004
2020	633 (19.4)	764 (18.8)	522 (19.1)	242 (18.3)	−0.020	−0.005
2021	581 (17.9)	804 (19.8)	509 (18.6)	295 (22.3)	0.089	0.007
2022	482 (14.8)	744 (18.3)	521 (19.0)	223 (16.9)	−0.058	−0.004
2023	211 (6.5)	364 (9.0)	253 (9.2)	111 (8.4)	−0.011	0.007

Abbreviations: ED, emergency department; IP, inverse probability; MOUD, medications for opioid use disorder; SMD, standardized mean difference; SROM, slow-release oral morphine; VCHA, Vancouver Coastal Health Authority.

^a^ Past year.

^b^ Score range, 0 to 26, with higher scores indicating more comorbidities.

^c^ Past 90 days.

### Statistical Analysis

We used descriptive statistics to summarize the baseline characteristics of the study cohort and inverse probability weighting, estimated using logistic regression, to adjust for potential baseline confounding in treatment assignment. To estimate the 12-month risk of treatment discontinuation and all-cause mortality in each arm, we used parametric G-computation^28^ and applied discrete time-survival analysis to person-day data (ie, time interval of 1 day). First, we fitted a weighted pooled logistic regression model that included treatment arm, time since baseline in days (natural cubic spline), interaction terms between treatment arm and cubic spline for time, baseline covariates (including year of MOUD episode initiation), and calendar month of MOUD episode initiation to account for potential seasonal effects. Second, we used the model’s estimated parameters to estimate the standardized cumulative incidence of each outcome across the full follow-up period as if the entire cohort had received SROM or methadone (eMethods 2 in Supplement 1 provides more details). The cumulative incidence, risk difference, and risk ratio at 12 months are presented. Nonparametric percentile bootstrap with 500 samples was used to compute the 95% CIs for the estimates. In the ITT model, person-trials received a time-fixed weight for baseline treatment assignment. The PP analysis included only individuals who received their assigned treatment within 4 days. These individuals received a weight equal to the product of the treatment assignment and treatment initiation weights at baseline to account for selection bias due to conditioning or restricting. Both weights were estimated in the entire eligible population, using logistic regression that included treatment arm, baseline covariates, and calendar month of MOUD episode initiation.

For the MOUD adherence (ie, PDC) outcome, we estimated the rate ratios for SROM vs methadone using negative binomial regression models with days receiving the assigned MOUD being the outcome, total number of follow-up days being the offset (365 days or mortality, whichever was earlier), and all covariates. Similar to the time-to-event analyses, the ITT model was weighted by baseline treatment assignment weight, while the PP model was weighted by the product of treatment assignment and treatment initiation weights. For ITT analysis, we used zero-inflated negative binomial regression to account for a high percentage (>10%) of complete nonadherence (ie, days on MOUD = 0).

We conducted several sensitivity analyses to evaluate the robustness and potential heterogeneity of our findings. First, we considered alternative discontinuation thresholds (ie, gaps of 14 and 30 days) based on previous studies.^29,30^ Second, given a number of shortages of the 24-hour formulation of SROM in BC occurring during the study period, we considered switches from this formulation (ie, Kadian) to the 12-hour version (ie, m-Eslon), as advised during these shortages, as being retained on SROM.^31^ Kadian shortages were identified from the Drug Shortages Canada and BC websites and confirmed with the BC Ministry of Health (eTable 6 in Supplement 1).^32,33^ Third, acknowledging that switches between MOUD are common, we also evaluated discontinuation from any MOUD. Fourth, we considered a shorter follow-up period (6 months), including the first eligible episode per 6-month period, to mimic the original RCT’s duration.^18^ Fifth, we randomly selected 1 episode per unique individual to evaluate the outcomes of multiple episodes per person. Sixth, we conducted an instrumental variable (IV) analysis using the prescriber’s past-6-month preference before the current prescription as an IV to adjust for potential unobserved factors influencing medication selection, consistent with the methods of prior studies in BC^34,35^ (eMethods 3 in Supplement 1 provides more details). Statistical significance was determined by a 95% CI excluding the null hypothesis value (eg, 0 for differences, 1 for ratios). All analyses were done using SAS, version 9.4 (SAS Institute Inc), and R, version 4.5.1 (R Project for Statistical Computing).

## Results

**Figure 1.**
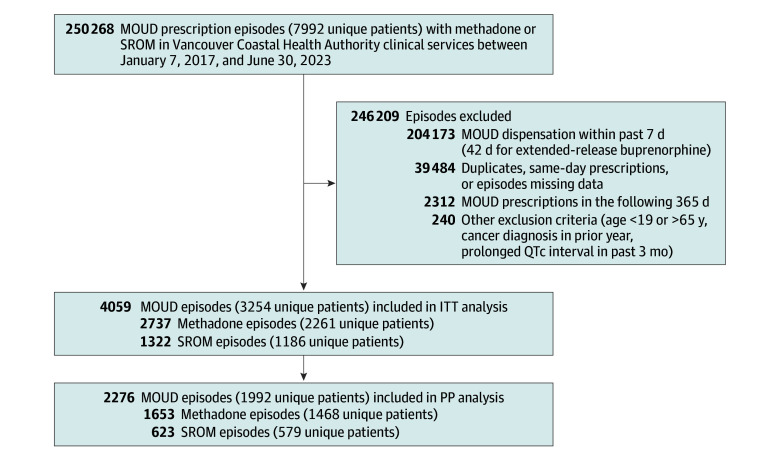
Flow Diagram of Cohort Construction ITT indicates intention to treat; MOUD, medications for opioid use disorder; PP, per protocol; QTc, corrected QT interval; SROM, slow-release oral morphine. Patients may have received both SROM and methadone treatment over time; thus, the sum of unique patients receiving each treatment exceeds the total number of unique patients in the ITT and PP analyses.

We identified 250 268 methadone- or SROM-based MOUD prescriptions among 7992 unique individuals during the study period. Of these, 3254 individuals met the eligibility criteria at least once, contributing to 4059 person-trials in the ITT sample (2737 [67.4%] in the methadone and 1322 [32.6%] in the SROM arm) (Figure 1). At baseline, the eligible individuals had a median age of 37 years (IQR, 30-46 years), were predominantly male (2088 [64.2%]; 1166 [35.8%] female), and were primarily from metropolitan or urban settings (3036 [93.3%]). Additionally, 1117 (34.3%) had a comorbid substance use disorder and 1195 (36.7%) had a comorbid psychiatric disorder (Table 1). MOUD initiations within 4 days of prescription (PP sample) occurred in 2276 person-trials (56.1%; 1653 [72.6%] in the methadone and 623 [27.4%] in the SROM arm) among 1992 unique individuals. Among methadone initiators, 241 of 1653 episodes (14.6%) had at least 1 take-home dose, with a median time to first take-home dose of 130 days (IQR, 52-230 days) and a median maximum number of consecutive doses of 2 (IQR, 2-3). Among SROM initiators, 168 of 623 episodes (27.0%) had at least 1 take-home dose, with a median of 94 days (IQR, 20-238 days) to first take-home dose and a median maximum of 3 (IQR, 2-7) consecutive doses.

Characteristics of the 4059 person-trials are presented in Table 1. The distribution of baseline characteristics was relatively well balanced across treatment groups, except that people in the methadone arm were more likely to have recent dispensation of opioid analgesics. Conversely, individuals in the SROM group were more likely to be older and have been codispensed sedative drugs and hydromorphone as safer supply.^27^ After applying inverse probability weighting, baseline covariates were balanced between treatment groups (all standardized mean differences <0.1).^36^

### ITT Analyses

**Figure 2.**
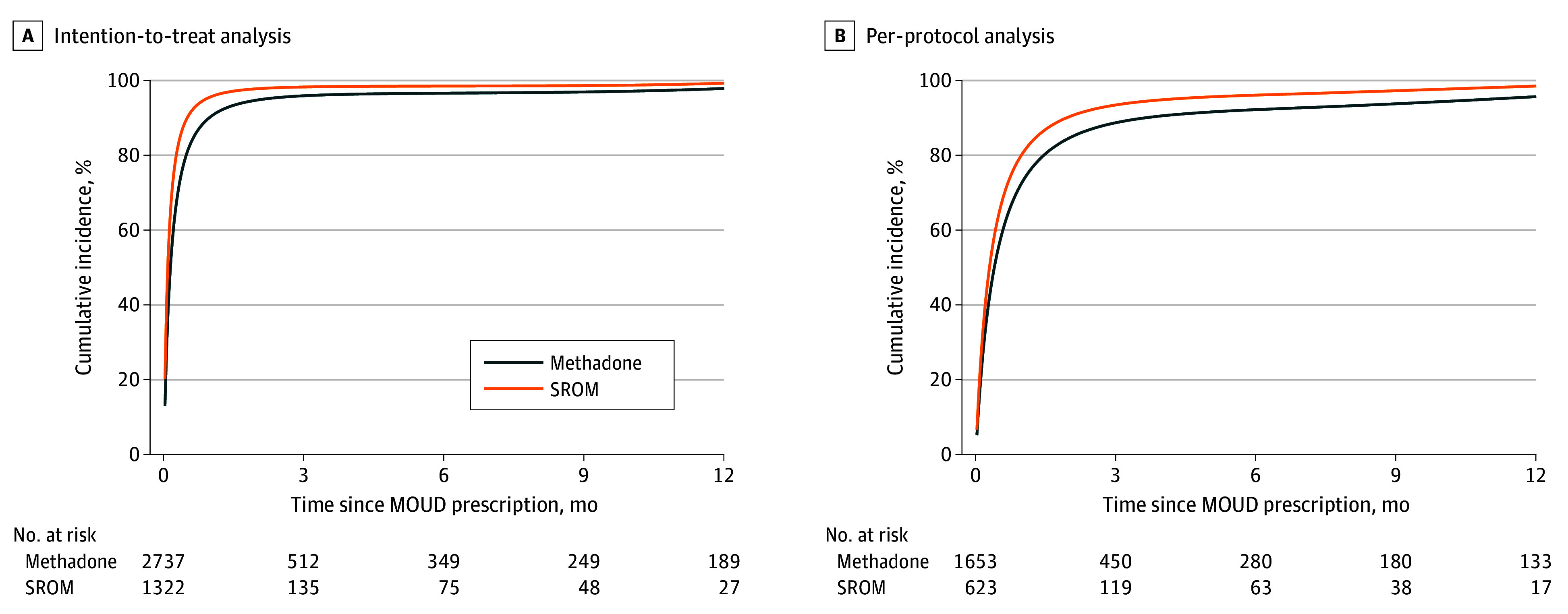
Survival Curves Showing Adjusted Cumulative Incidence of Assigned-Treatment Discontinuation, Stratified by Treatment Arm MOUD indicates medications for opioid use disorder; SROM, slow-release oral morphine. Adjustment factors included sociodemographic variables, medical history, health care utilization, concomitant medications in the past 90 days, and prescription episode characteristics.

The median duration of treatment was 8 days (IQR, 1-51 days) days in the methadone group and 3 days (IQR, 1-23 days) days in the SROM group, with a standardized cumulative rate of discontinuation at 12 months of 97.9% (95% CI, 97.5%-98.2%) and 99.3% (95% CI, 99.0%-99.5%), respectively (Figure 2A). The adjusted risk difference (ARD) between the SROM and methadone arms was 1.4 percentage points (pp) (95% CI, 1.0-1.9 pp), yielding a 12-month adjusted risk ratio for discontinuation of 1.01 (95% CI, 1.01-1.02) for the SROM arm compared with the methadone arm (Table 2). Mean adherence rate (ie, PDC) to the assigned MOUD during the 12 months of treatment was also lower for the SROM group (29.3%; 95% CI, 28.3%-30.5%) than for the methadone group (33.0%; 95% CI, 32.0%-34.0%), corresponding to an adjusted risk ratio of 0.89 (95% CI, 0.87-0.91) (Table 2 and the eFigure in Supplement 1).

**Table 2.** Adjusted Primary and Secondary Outcomes at 12 Months by Treatment Arm Using ITT and PP Approaches

Outcome	Cumulative incidence or mean rate, % (95% CI)		SROM vs methadone	
	Methadone	SROM	ARD, pp (95% CI)^a^	ARR (95% CI)^a^
**Treatment discontinuation, incidence**				
ITT	97.9 (97.5-98.2)	99.3 (99.0-99.5)	1.4 (1.0 to 1.9)	1.01 (1.01-1.02)
PP	95.7 (94.9-96.4)	98.5 (97.8-99.1)	2.8 (2.0 to 3.7)	1.03 (1.02-1.04)
**All-cause mortality, incidence**				
ITT	7.0 (5.3-8.6)	8.2 (6.1-10.6)	1.1 (−1.3 to 4.1)	1.18 (0.82-1.69)
PP	6.2 (4.4-8.4)	5.5 (3.0-8.4)	−0.6 (−4.0 to 2.8)	0.89 (0.48-1.52)
**Adherence (PDC), rate**				
ITT	33.0 (32.0-34.0)	29.3 (28.3-30.5)	−3.7 (−4.9 to −3.0)	0.89 (0.87-0.91)
PP	42.6 (41.1-44.2)	36.9 (35.2-38.5)	−5.6 (−6.8 to −4.6)	0.87 (0.84-0.89)

Abbreviations: ARD, adjusted risk difference; ARR, adjusted risk ratio; ITT, intention to treat; PDC, proportion of days covered; PP, per protocol; pp, percentage points; SROM, slow-release oral morphine.

^a^ Adjustment factors included sociodemographic variables, medical history, health care utilization, concomitant medications in the past 90 days, and prescription episode characteristics.

**Figure 3.**
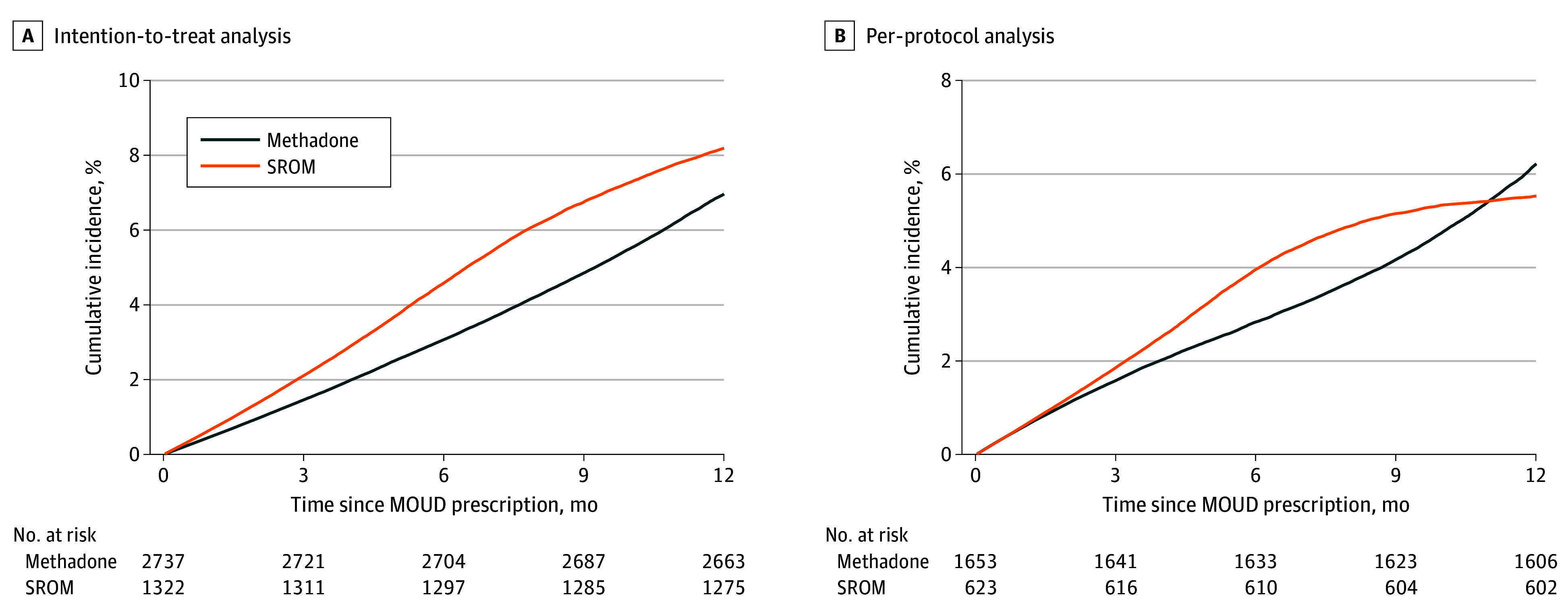
Survival Curves Showing Adjusted Cumulative Incidence of All-Cause Mortality, Stratified by Treatment Arm MOUD indicates medications for opioid use disorder; SROM, slow-release oral morphine. Adjustment factors included sociodemographic variables, medical history, health care utilization, concomitant medications in the past 90 days, and prescription episode characteristics.

There were 121 deaths over follow-up: 47 (40 per 1000 person-years) in the SROM arm and 74 (33 per 1000 person-years) in the methadone arm. Around half of these deaths (n = 68 [56.2%]) were overdose related (opioids were involved in 66 deaths [97.1%]), and all except 2 deaths (1.7%) occurred when people were not receiving treatment. The adjusted cumulative incidence of all-cause mortality at 12 months of follow-up was 8.2% (95% CI, 6.1%-10.6%) for the SROM group and 7.0% (95% CI, 5.3%-8.6%) for the methadone group (Figure 3A), with no significant differences between the 2 groups (ARD, 1.1 pp [95% CI, −1.3 to 4.1 pp]; adjusted risk ratio, 1.18 [95% CI, 0.82-1.69]) (Table 2).

### PP Analyses

When only considering person-trials in which patients actually started their prescribed MOUD (n = 2276), treatment duration increased in both groups: median of 26 days (IQR, 8-121 days) for methadone vs 19 days (IQR, 7-68 days) for SROM. The adjusted 12-month cumulative incidence of treatment discontinuation was 98.5% (95% CI, 97.8%-99.1%) for the SROM arm and 95.7% (95% CI, 94.9%-96.4%) for the methadone arm, corresponding to a higher risk of discontinuation in the SROM arm (ARD, 2.8 pp; 95% CI, 2.0-3.7 pp) (Table 2).

In the PP analysis, adjusted mean adherence rates were higher (42.6% [95% CI, 41.1%-44.2%] for methadone and 36.9% [95% CI, 35.2%-38.5%] for SROM). However, SROM remained associated with lower adherence than methadone (adjusted risk ratio, 0.87; 95% CI, 0.84-0.89) (Table 2).

The PP all-cause mortality results were also consistent with the ITT analyses. There were 21 deaths (36 per 1000 person-years) in the SROM arm and 47 deaths (32 per 1000 person-years) in the methadone arm. The adjusted cumulative incidence of all-cause mortality at 12 months of follow-up was 5.5% (95% CI, 3.0%-8.4%) for the SROM group and 6.2% (95% CI, 4.4%-8.4%) for the methadone group (Figure 3B), with no significant difference between arms (ARD, −0.6 pp; 95% CI, −4.0 to 2.8 pp) (Table 2).

### Sensitivity Analysis

Sensitivity analyses yielded similar results for the discontinuation outcome (eTable 7 in Supplement 1). Of note, the IV approach to control for patient-level indication found a slightly larger ARD than the primary PP analysis (6.0 pp [95% CI, 3.2-8.9 pp] vs 2.8 pp [95% CI, 2.0-3.7 pp]), but results were consistent in direction.

## Discussion

In this emulated trial using population-level observational data including 4059 OUD treatment episodes from VCHA clinical services in the fentanyl era, treatment discontinuation and nonadherence were common for both SROM and methadone. However, in both the ITT and PP analyses, SROM was associated with slightly higher risk of treatment discontinuation (1.4-pp absolute increase) and more than 10% lower PDC (ie, adherence) than methadone during the 12-month follow-up period. Sensitivity analyses demonstrated similar results with limited impact when allowing for medication switches and varying the outcome definition. There were no observed significant differences between the 2 treatments in all-cause mortality risk.

A recent comparative effectiveness study evaluating more than 90 000 treatment episodes with methadone and buprenorphine-naloxone in BC between 2010 and 2020 found a 12-month cumulative incidence of methadone discontinuation of more than 80%.^35^ Findings from our analysis demonstrated similarly low methadone (and SROM) retention rates, with a 12-month discontinuation rate of more than 95% and a mean PDC of less than 40%. The worse indicators observed in our study may be attributable to study design differences. Specifically, while our discontinuation definition was similar to the study by Nosyk et al,^35^ their study population included only people who actually initiated MOUD. In addition, our study period was restricted to 2017 to 2024, when fentanyl and other high-potency synthetic opioids were highly prevalent in the unregulated drug supply, which alongside the COVID-19 pandemic created new challenges for the treatment of OUD.

Unlike previous RCTs conducted in Europe,^37,38,39^ our study found overall low retention rates and a small but statistically significant advantage of methadone over SROM for treatment retention. Differences in our study’s results compared with previous European RCTs may be explained by a number of factors. First, our study adopted an emulated trial approach using population-level observational data derived from an actual clinical setting. Our study’s larger sample size allowed for greater statistical power to detect significant differences. Also, clinical data inherently differ from data collected in traditional RCTs, including less risk of self-selection bias and artificial inflation of retention rates sometimes observed in RCTs due to common financial compensation.^40^ Additionally, differences in study context, including differences in access to MOUD, and the highly toxic drug supply prevalent in Vancouver during the study period may further explain the discrepant findings. People who use fentanyl typically require higher MOUD doses than what has been previously reported for people who use heroin or other opioids.^41,42^ This, in turn, makes stabilization with MOUD more challenging, with the added difficulty of managing symptoms associated with contaminants in the unregulated drug supply (eg, benzodiazepines, xylazine). It may also be the case that as SROM is a newer treatment option, health care practitioners’ lower familiarity with and experience prescribing SROM may have contributed to the lower rate of retention on treatment compared with methadone. The slightly higher risk difference for discontinuation for SROM compared with methadone observed in the IV analysis further suggests prescriber practice may be a key factor in medication selection, perhaps owing to less experience with SROM as a newer agent compared with methadone. In addition, the observation that, compared with individuals in the methadone arm, those in the SROM arm were more likely to have received hydromorphone as a safer supply, a policy initiative implemented during the COVID-19 pandemic to reduce dual exposure to both the toxic drug supply and COVID-19,^27^ suggests that individuals who were prescribed SROM may have had more complex OUD and therefore may have been at increased risk of poorer treatment outcomes than those who were prescribed methadone.

In addition, although the risk of all-cause mortality was high in both groups, it was comparable with all-cause mortality rates for people with OUD in this and other settings.^43,44,45,46^ Of importance, we did not find a statistically significant difference in mortality risk between the 2 treatment arms, and almost all of the deaths occurred when people were not engaged in treatment, underscoring the importance of continuous engagement in MOUD.

### Strengths and Limitations

Our study has strengths. To our knowledge, our study is the first to provide clinical evidence on the comparative effectiveness of SROM and methadone for the treatment of OUD in a setting with high prevalence of high-potency synthetic opioids. Another strength of our study is that we had access to prescription data via patient EMRs, allowing us to emulate the randomization event of an RCT (ie, at the time of a clinical encounter where a prescription was given), potentially improving the validity of our ITT analysis results.

Our study also has some limitations. First, the study population was limited to people seen in VCHA clinics, which are mostly located in urban settings and where health care is publicly funded, including MOUD coverage under BC PharmaCare plans. As such, our results may not be generalizable to more rural areas or jurisdictions with different health system arrangements. Second, unmeasured confounding of variables not captured in our health administrative data may bias our results, even after adjusting for known demographic and clinical factors that affect MOUD choice and engagement in treatment. For example, patients’ preferences and how they aligned with the physicians’ prescribing were not captured in our data. Furthermore, time-varying confounders (eg, adequate dosing) were not considered in our analysis. Although an RCT design could overcome some of these limitations, given that both medications are a standard part of clinical care in our setting, one characterized by high rates of switching between the 2 medications, an RCT would have other important limitations, including its reduced representativeness of the clinical cohort seeking OUD care in our study’s setting. Third, despite our attempt to account for shortages in the 24-hour SROM formulation, there may have been additional disruptions to OUD care during these shortages that may have influenced our results.^47^ Fourth, our analysis did not explicitly account for out-of-province migration. However, because migration patterns are unlikely to differ substantially between treatment groups, any misclassification of migration as treatment discontinuation or nonadherence is expected to be nondifferential and unlikely to meaningfully bias our comparative estimates.

## Conclusions

Findings from this study suggest that SROM may be associated with small absolute differences in but comparatively higher risks of treatment discontinuation than methadone in clinical settings contextualized by a toxic drug supply and low overall initiation and retention rates on MOUD. Despite the inferiority of SROM compared with methadone for treatment continuity and adherence, there was no difference in mortality risk. It may still be appropriate to select either medication based on patient factors and preferences within a patient-centered care framework that emphasizes treatment engagement in decision-making, as long as measures are in place to support retention.^48,49,50^

## Notes

Supplement 1. **eTable 1.** Key Protocol Components of the Original pRESTO RCT and the Emulated Trial **eTable 2.** Administrative Health Data Sources **eTable 3.** DIN/PINs for Identification of Medications for Opioid Use Disorder From PharmaNet **eTable 4.** *ICD-9* and *ICD-10* Codes Used to Identify Eligibility Criteria and Potential Confounders **eTable 5.** AHFS/ATC Classification Numbers Used for the Identification of Dispensed Medications From PharmaNet **eTable 6.** Reported Kadian Shortages in British Columbia, 2017-2024 **eTable 7.** Adjusted Cumulative Incidence, Risk Differences, and Risk Ratios of the Primary Outcome (Treatment Discontinuation) in Sensitivity Analyses **eMethods 1.** MOUD Episode Construction **eMethods 2.** Cumulative Incidence Standardization **eMethods 3.** Instrumental Variable Analysis **eFigure.** Adherence to Medications for Opioid Use Disorder (MOUD) Over 365 Days (3997 Episodes)
eReferences

Supplement 2.
Data Sharing Statement
