# Supplementary material for: Slow-Release Oral Morphine vs Methadone for Opioid Use Disorder in the Fentanyl Era
Source: JAMA Netw Open. 2026 Mar 24;9(3):e262970. doi: 10.1001/jamanetworkopen.2026.2970 (PMC13014207; doi:10.1001/jamanetworkopen.2026.2970)
Supplement: Supplement 1. — eTable 1. Key Protocol Components of the Original pRESTO RCT and the Emulated Trial eTable 2. Administrative Health Data Sources eTable 3. DIN/PINs for Identification of Medications for Opioid Use Disorder From PharmaNet eTable 4. ICD-9 and ICD-10 Codes Used to Identify Eligibility Criteria and Potential Confounders eTable 5. AHFS/ATC Classification Numbers Used for the Identification of Dispensed Medications From PharmaNet eTable 6. Reported Kadian Shortages in British Columbia, 2017-2024 eTable 7. Adjusted Cumulative Incidence, Risk Differences, and Risk Ratios of the Primary Outcome (Treatment Discontinuation) in Sensitivity Analyses eMethods 1. MOUD Episode Construction eMethods 2. Cumulative Incidence Standardization eMethods 3. Instrumental Variable Analysis eFigure. Adherence to Medications for Opioid Use Disorder (MOUD) Over 365 Days (3997 Episodes) eReferences [file jamanetwopen-e262970-s001.pdf]

Supplementary Online Content

Socias ME, Lei J, Zhou V, et al. Slow-release oral morphine vs methadone for opioid use disorder in the fentanyl era. *JAMA Netw Open*. 2026;9(3):e262970. doi:10.1001/jamanetworkopen.2026.2970

eTable 1. Key Protocol Components of the Original pRESTO RCT and the Emulated Trial

eTable 2. Administrative Health Data Sources

eTable 3. DIN/PINs for Identification of Medications for Opioid Use Disorder From PharmaNet

eTable 4. *ICD-9* and *ICD-10* Codes Used to Identify Eligibility Criteria and Potential Confounders

eTable 5. AHFS/ATC Classification Numbers Used for the Identification of Dispensed Medications From PharmaNet

eTable 6. Reported Kadian Shortages in British Columbia, 2017-2024

eTable 7. Adjusted Cumulative Incidence, Risk Differences, and Risk Ratios of the Primary Outcome (Treatment Discontinuation) in Sensitivity Analyses

eMethods 1. MOUD Episode Construction

eMethods 2. Cumulative Incidence Standardization

eMethods 3. Instrumental Variable Analysis

eFigure. Adherence to Medications for Opioid Use Disorder (MOUD) Over 365 Days (3997 Episodes)

eReferences

This supplementary material has been provided by the authors to give readers additional information about their work.

© 2026 Socias ME et al. *JAMA Network Open*.

eTable1. Key protocol components of the original pRESTO RCT and the emulated trial

Component	Target Trial (original pRESTO study)	Emulated Trial
Aim	To evaluate the comparative effectiveness of SROM-based vs. methadone for the treatment of OUD.	
Data	Prospective 24 Week RCT. Sample size of 198.	Administrative linked retrospective cohort of people with diagnosed OUD seen in community clinics in VCHA between 2018 and 2024.
Eligibility	Inclusion:<br>• Diagnosed with OUD requiring MOUD, as per DSM-5 criteria.<br>• 19 and 65 years of age, inclusively.<br>• Willing and eligible to be randomized to SROM or methadone-based MOUD.<br>• Be of non-childbearing potential or willing to use an acceptable method of contraception.<br>• negative pregnancy test at screening and thereafter.<br>• Be able to provide written informed consent.<br>• Be able to communicate in English.<br><br>Exclusion:<br>• Any disabling, severe, or unstable medical or psychiatric condition that, in the opinion of the study physician, precludes safe participation in the study<br>• Any severe or unstable co-morbid substance use disorder<br>• Maintenance on buprenorphine methadone slow-release oral morphine at doses of ≥4 mg in the 5 days prior to screening<br>• Females who are pregnant.<br>• History of a serious adverse drug reaction, hypersensitivity reaction, or allergy to methadone or SROM.<br>• Use of an investigational drug in the 30 days prior to screening.<br>• Pending legal action or other reasons that might prevent completion of the study.<br>• Current or anticipated need for treatment with any medication that may interact with methadone or SROM	Inclusion:<br>• Individuals with OUD as per MOUD prescription or an ICD 9/10 code for OUD.<br>• 19 and 65 years of age, inclusively.<br>• Newly prescribed or re-prescribed treatment with (1) SROM or (2) methadone (i.e., no MOUD dispensations in the past 7 days) between 1 July 2018 and 30 June 2023<br><br>Exclusion:<br>• Prolonged QTc.<br>• Individuals with cancer.<br>• Prescribed more than one MOUD on the same day.
Intervention strategies	1. SROM dispensed under daily witnessed ingestion.<br>2. Methadone as daily witnessed ingestion.	1. New SROM prescription<br>2. New methadone prescription
Treatment assignment	1:1 Randomisation	Prescription of to one or other MOUD as per EMR data. Baseline characteristics between

© 2026 Socias ME et al. *JAMA Network Open*.

		arms balanced using propensity score weighting
Follow-up	Starts on the day of randomization and ends 24 weeks after treatment initiation.	Starts on day of the prescription until end of the 365-day study period.
Outcomes	Primary: suppression of illicit opioid use (overall percentage of opioid-free UDT).<br><br>Secondary: treatment retention, safety, overdose events, treatment satisfaction, psychological functioning, changes in drug related problems, changes in quality of life, opioid cravings, other substance use, and cost-effectiveness.	Primary: treatment discontinuation.<br><br>Secondary: adherence, all-cause mortality, illicit opioid use, overdose events, acute care utilization.
Casual contrast	ITT and PP	Analog of ITT and PP effects
Statistical analysis	The sample size calculation is based on assumptions for continuous outcomes in parallel group non-inferiority studies, using a non-inferiority margin of 10%. For analysis of the main outcome, non-inferiority will be assessed calculating the 95% Confidence Interval (CI) of the mean difference in the proportion of opioid-free urines between the 2 arms. Non-inferiority will be demonstrated if the lower limit of the 95% CI lies above -10%. Analyses for secondary outcomes will use "superiority" hypotheses, where two-sided tests will be performed with a significance level of 5%.	Weighted pooled logistic regression model that included treatment arm, time in days (natural cubic spline terms), baseline covariates, and calendar month of MOUD episode initiation to account for potential seasonal effects. The ITT model was only weighted by treatment assignment weights. The final weight for the PP analysis was constructed by multiplying the treatment assignment weight by the treatment initiation weight.

© 2026 Socias ME et al. *JAMA Network Open*.

eTable 2: Administrative health data sources

Data source*	Description
Vancouver Coastal Health Electronic Medical records	Demographics, indication of OUD diagnosis, MOUD prescription
PharmaNet (PNET)	Records of all prescription drug dispensations in community pharmacies in BC.
Medical Service Plan (MSP)	Records of all fee-for-service provider visits billed to BC's public health insurance program
Discharge Abstract Database (DAD)	Records of all stays in acute care hospitals in BC.
National Ambulatory Care Reporting System (NACRS)	Records on ambulatory care within BC, covering 67% of ED visiting in the province and almost 100% of emergency department visits in the two urban health authorities (i.e., VCH and Fraser).
Vital Statistics	All death and their underlying causes registered in BC
Muse	ECG data, including QTc interval measurement.

* Databases were linked using the client's unique personal identifier

Access to data provided by the Data Stewards is subject to approval but can be requested for research projects through the Data Stewards or their designated service providers. The following data sets were used in this study are presented in eTable1. You can find further information regarding these data sets by visiting the PopData project webpage at: *https://my.popdata.bc.ca/project_listings/22-101*
All inferences, opinions, and conclusions drawn in this publication are those of the author(s), and do not reflect the opinions or policies of the Data Steward(s).

© 2026 Socias ME et al. *JAMA Network Open*.

eTable 3. DIN/PINs for identification of medications for opioid use disorder from PharmaNet*

MOUD	DIN/PIN
Methadone	999792, 999793, 66999990, 66999991, 66999992, 66999993, 66999997, 66999998, 66999999, 67000000, 67000001, 67000002, 67000003, 67000004, 67000005, 67000006, 67000007, 67000008, 67000009,67000010, 67000011, 67000012, 67000013, 67000014, 67000015, 67000016, 67000017, 67000018, 67000019, 67000020
Slow-release oral morphine (24-hour formulation)	22123349, 22123346, 22123347, 22123348
Buprenorphine/naloxone	2295695, 2295709, 2408090, 2408104, 2424851, 2424878, 2453908, 2453916, 2468085, 2468093
Injectable diacetylmorphine or hydromorphone	66123367, 2146126, 22123340
Extended-release buprenorphine	02483084, 02483092
MOUD, medication for opioid use disorder. DIN, Drug Identification Number. PIN, Product Identification Number.	

*Adapted from Nosyk et al.[1]

© 2026 Socias ME et al. *JAMA Network Open*.

eTable 4. *ICD-9 and ICD-10* codes used to identify eligibility criteria and potential confounders

Eligibility criteria/ Comorbidity	ICD-9 (DAD/MSP)	ICD-10 (DAD/NACRS)
Opioid use disorder	304.0, 304.7, 305.5	F11
Cancer	140-208	C00-C97
Chronic pain	338.2, 338.4, 307.80, 307.89, 338.0, 719.41, 719.45-719.47, 719.49, 720.0, 720.2, 720.9, 721.0-721.4, 721.6, 721.8, 721.9, 722, 723.0, 723.1, 723.3-723.9, 724.0-724.6, 724.70, 724.79, 724.8, 724.9, 729.0-729.2, 729.4, 729.5, 350, 352-357, 344.0, 344.1, 997.0, 733.0, 733.7, 733.9, 781	F45.4, G89.0, G89.2, G89.4, M08.1, M25.50, M25.51, M25.55-M25.57, M43.2-M43.6, M45, M46.1, M46.3, M46.4, M46.9, M47, M48.0, M48.1, M48.8, M48.9, M50.8, M50.9, M51, M53.1-M53.3, M53.8, M53.9, M54, M60.8, M60.9, M63.3, M79.0-M79.2, M79.6, M79.7, M96.1, G50, G52-G64, G82, G97, M89, R29
Hepatitis C virus	070.41, 070.44, 070.51, 070.54, 070.70, 070.71, V02.62	B17.10, B17.11, B18.2, B19.20, B19.21, Z22.52
Non-fatal opioid overdose	965.0, E850.0, E850.1, E850.2	T40.0X1, T40.0X2, T40.0X4, T40.0X5, T40.1X1, T40.1X2, T40.1X4, T40.2X1, T40.2X2, T40.2X4, T40.2X5, T40.3X1, T40.3X2, T40.3X4, T40.3X5, T40.4X1, T40.4X2, T40.4X4, T40.4X5
Alcohol use disorder[a]	291, 303, 305.0, 357.5, 425.5, 535.3, 571.0-571.3	F10, G31.2, G62.1, G72.1, I42.6, K29.2, K70, K86.0
Stimulant use disorder	3042, 3056, 3044, 3057	3042, 3056, 3044, 3057
Sedative use disorder	3041, 3054	F13
Serious mental disorder (e.g., schizophrenia, bipolar disorder)	295, 297, 298, 296.0, 296.1, 296.4-9	F20-29, F31
Less serious mental disorder (e.g., depressive and anxiety disorders)	296.2, 296.3, 300.4, 311, 300.0, 300.2, 300.3, 309.81	F32, F33, F34.1, F43.21, F43.23, F40, F41, F42, F43.22, F43.1, F43.12, F43.23
Homelessness	V60.0; V60.1	Z59.0, Z59.1

DAD, Discharge Abstract Database. MSP, Medical Service Plan. NACRS, National Ambulatory Care Reporting System.
[a] Algorithm also included ATC for naltrexone (N07BB04), acamprosate (N07BB03) and disulfiram (N07BB01) from Pharmanet

- **Injection drug use** (yes vs. no): based on a validated algorithm using MSP and DAD data and refers to either 2 primary care visits (MSP) or 1 hospitalization (DAD) for injectable drugs in the past 1 year.[2]
- **Region of residence**: stratified by Health Authority area, using client's postal code, and dichotomized at Vancouver Coastal vs others (Fraser, Interior, Island, Northern).
- **Degree of urbanicity of region of residence**: (Metro/urban vs. rural/remote): Categorized by local health authority (LHA) based on population (>/≤ 40 000) and geographical consideration (proximity to a larger population center and health services), using client's postal code.[3]
- **Modified Elixhauser comorbidity score** (≥2, 1 vs. 0) was derived from DAD and was modified by excluding ICD-10 codes for substance use and mental disorders given that these were assessed separately.[4,5]
- **Attachment to a regular health provider** (yes vs. no): based on whether a client had ≥ 3 primary care visits (MSP) in the past year, and ≥50% visits were with the same provider. If <50% those visits were with the same provider or <3 primary care visits, categorized as "no".[1]

- **Low-income status:** BC PharmaCare plan C/G (i.e., for individuals with an annual income less than $42,000) as per PharmaNet.

© 2026 Socias ME et al. *JAMA Network Open*.

eTable 5. AHFS/ATC classification numbers used for the identification of dispensed medications from PharmaNet

Medication class	Codes
Sedative medications	Benzodiazepines: AHFS 28:24.08<br>Z-drugs: AHFS 28:24:92<br>Gabapentinoids: ATC N03AX12, N03AX16
Non-sedative psychiatric medications	Antidepressants: 28:16.04.xx<br>Antipsychotics: AFHS: 28:16.08.xx
Opioid analgesics	ATC: N02A (except when PIN denoted use as OAT)
AHFS, American Hospital Formulary Service. ATC, Anatomical Therapeutic Chemical.	

© 2026 Socias ME et al. *JAMA Network Open*.

eTable 6. Reported Kadian shortages in British Columbia, 2017-2024

Shortage start (Month/year)	Shortage end (Month/year)	Dose (mg)
Jan 10, 2018	Mar 26, 2018	100
Jan 10, 2018	Feb 17, 2018	50
Jan 10, 2018	Feb 17, 2018	50
Feb 21, 2018	Mar 21, 2018	50
Oct 21, 2019	Dec 21, 2019	50
Oct 21, 2019	Dec 21, 2019	20
Oct 21, 2019	Dec 21, 2019	100
Nov 25, 2019	Jan 12, 2020	100
Dec 13, 2019	May 21, 2020	50
Dec 13, 2019	Mar 12, 2020	20
Dec 13, 2019	Feb 15, 2020	100
Mar 02, 2020	Apr 10, 2020	100
Mar 02, 2020	Apr 04, 2020	20
Mar 11, 2020	May 02, 2020	100
Mar 24, 2020	Jul 18, 2020	20
Mar 02, 2021	Apr 30, 2021	100
Mar 02, 2021	Apr 11, 2021	50
Mar 15, 2021	May 07, 2021	50
Mar 15, 2021	Apr 30, 2021	20
Apr 22, 2021	Jun 27, 2021	100
Jul 05, 2021	Sep 03, 2021	50
Jul 05, 2021	Aug 30, 2021	100

To simplify analysis, episodes with a start date within 30 days of another episode were clustered together resulting in the following three discrete Kadian shortage episodes

Episode	Shortage start	Shortage end
1	Jan 10, 2018	Mar 26, 2018
2	Oct 21, 2019	Jul 18, 2020
3	Mar 02, 2021	Sep 03, 2021

© 2026 Socias ME et al. *JAMA Network Open*.

eTable 7. Adjusted cumulative incidence, risk differences, and risk ratios of the primary outcome (treatment discontinuation) in sensitivity analyses

Outcome	Cumulative incidence, % (95% CI)		SROM vs Methadone	
	Methadone	SROM	Risk difference, % (95% CI)	Risk ratio (95% CI)
Discontinuation: 14 days				
ITT	96.9 (96.4, 97.3)	98.7 (98.4, 99.1)	1.9 (1.4, 2.4)	1.02 (1.01, 1.03)
PP	93.6 (92.4, 94.6)	97.0 (96.1, 98.0)	3.4 (2.1, 4.8)	1.04 (1.02, 1.05)
Discontinuation: 30 days				
ITT	95.6 (94.9, 96.1)	98.1 (97.6, 98.5)	2.5 (1.8, 3.2)	1.03 (1.02, 1.03)
PP	90.7 (89.2, 91.8)	94.9 (93.6, 96.2)	4.3 (2.6, 5.9)	1.05 (1.03, 1.07)
Allowing for switches from Kadian to m-Eslon (during Kadian shortages)				
ITT	97.3 (97.0, 97.5)	98.4 (98.2, 98.6)	1.1 (0.8, 1.4)	1.01 (1.01, 1.02)
PP	95.2 (94.7, 95.7)	98.1 (97.7, 98.5)	2.9 (2.3, 3.5)	1.03 (1.02, 1.04)
Allowing for any MOUD switch				
ITT	97.4 (97.0, 97.8)	98.9 (98.6, 99.2)	1.5 (1.1, 1.9)	1.02 (1.01, 1.02)
PP	94.7 (93.9, 95.5)	97.9 (97.0, 98.6)	3.2 (2.1, 4.2)	1.03 (1.02, 1.04)
Follow-up period: 6 months				
ITT	97.1 (96.7, 97.5)	99.0 (98.7, 99.2)	1.9 (1.5, 2.3)	1.02 (1.02, 1.03)
PP	94.1 (93.2, 94.9)	98.0 (97.3, 98.5)	3.9 (3.0, 4.9)	1.04 (1.03, 1.05)
Randomly select one episode per unique ID				
ITT	97.7 (97.2, 98.1)	99.3 (99.0, 99.6)	1.6 (1.1, 2.1)	1.02 (1.01, 1.02)
PP	95.2 (94.3, 96.1)	98.5 (97.6, 99.1)	3.3 (2.2, 4.3)	1.03 (1.02, 1.05)
Prescriber-preference IV approach				
PP (CACE)	88.7 (85.0, 92.4)	94.7 (91.6, 97.8)	6.0 (3.2, 8.9)	1.07 (1.05, 1.09)

CI, confidence interval. SROM, slow-release oral morphine. ITT, Intention-to-Treat. PP, Per-protocol. IV, instrumental variable. CACE, complier average causal effect.
Note: The IV approach estimated counterfactual outcomes as if all were treated with SROM vs methadone. Because estimates are derived from the same individuals, arm CIs may overlap even when RD/RR CIs are significant.

© 2026 Socias ME et al. *JAMA Network Open*.

eMethods 1. MOUD episode construction

MOUD episodes were constructed using the “service date” and “days of supply” fields in PharmaNet. The MOUD episode began on the “service date” and the length was calculated as the difference between the last and first day of dispensed medication, as long as there were no gaps in prescribed doses lasting >7 days. Given that we did not have access to medication information during hospitalization, it was assumed that individuals who were on MOUD at the time of hospitalization, continued the same MOUD and dose during the inpatient stay.

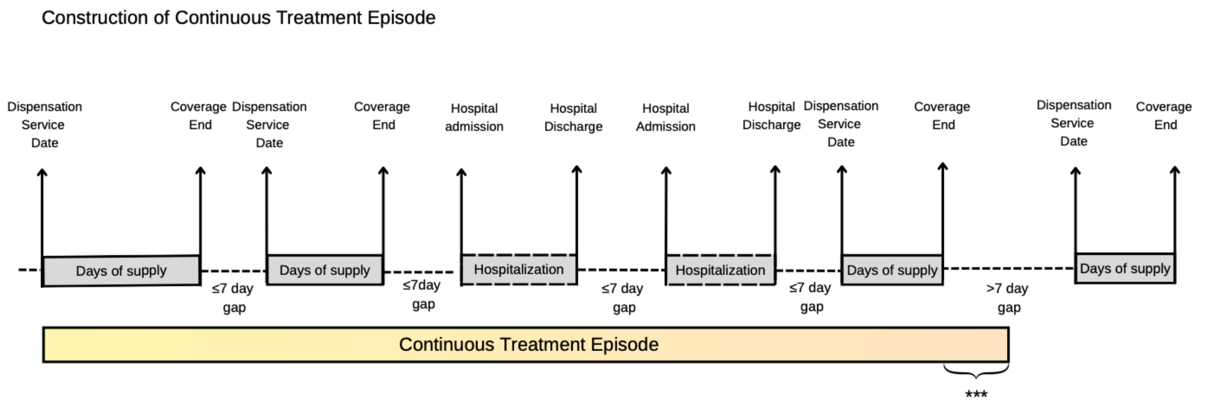

*** For the discontinuation outcome, end of episode was defined as coverage end + 1 day of the last dispensation before a >7-day gap. For the mortality outcome, the end of episode was defined as coverage end + 7 days.

© 2026 Socias ME et al. *JAMA Network Open*.

eMethods 2. Cumulative incidence standardization:

After fitting the weighted pooled logistic regression model for the outcome, we used the model estimates to generate counterfactual, subject-specific predicted probabilities of the outcome at each time interval under the two treatment strategies: (1) as if all individuals in the cohort had received SROM and (2) as if all individuals had received methadone. These predicted probabilities were interpreted as discrete-time hazards. For each individual and treatment strategy, we converted the predicted hazards into survival probabilities over the full follow-up period. Finally, we derived the cumulative incidence of outcome as one minus the survival function, as well as the corresponding risk difference and risk ratio. The cumulative incidence, risk difference, and risk ratio at 12 months were presented. Non-parametric percentile bootstrap with 500 samples was used to compute the 95% confidence intervals for the estimates.

© 2026 Socias ME et al. *JAMA Network Open*.

eMethods 3. Instrumental variable analysis

We adopted an IV approach to control for patient-level indication and approximate a comparison in patients who were clinically eligible for either drug but received one medication due to prescriber preference (i.e., compliers). Given that prescriber information was only available in PharmaNet (i.e., on dispensed medications), this approach was only considered for the PP population. In this IV analysis, we estimated the complier average causal effect (CACE) via a two-stage residual inclusion approach. We included a prescriber-level random intercept in the first-stage treatment model to account for within-prescriber clustering of treatment decisions, and used the same patient-level clustered bootstrap approach to account for individual-level correlation.

© 2026 Socias ME et al. *JAMA Network Open*.

eFigure. Adherence to medications for opioid use disorder (MOUD) over 365 days (3997 episodes)

Each row represent data for one episode, with daily adherence being plotted along the x axis. Episodes are ordered by the percentage of adherence to the assigned MOUD (green), then to the any MOUD (yellow), then to non-adherence (orange). Grey indicates missing data (censoring) due to death (black).

A- Slow-release oral morphine (1322 episodes)

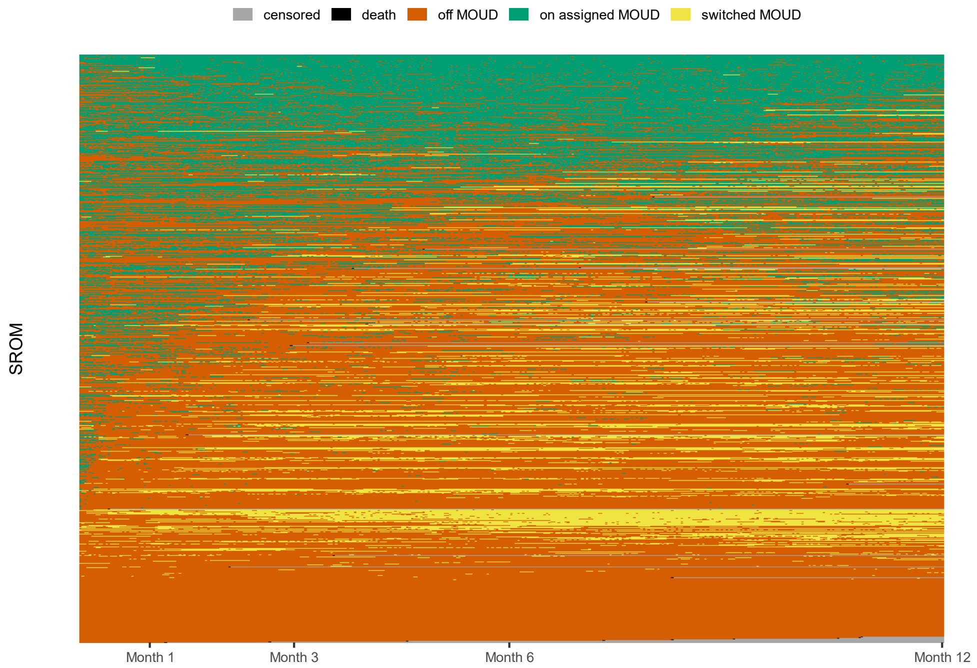

B- Methadone (2737 episodes)

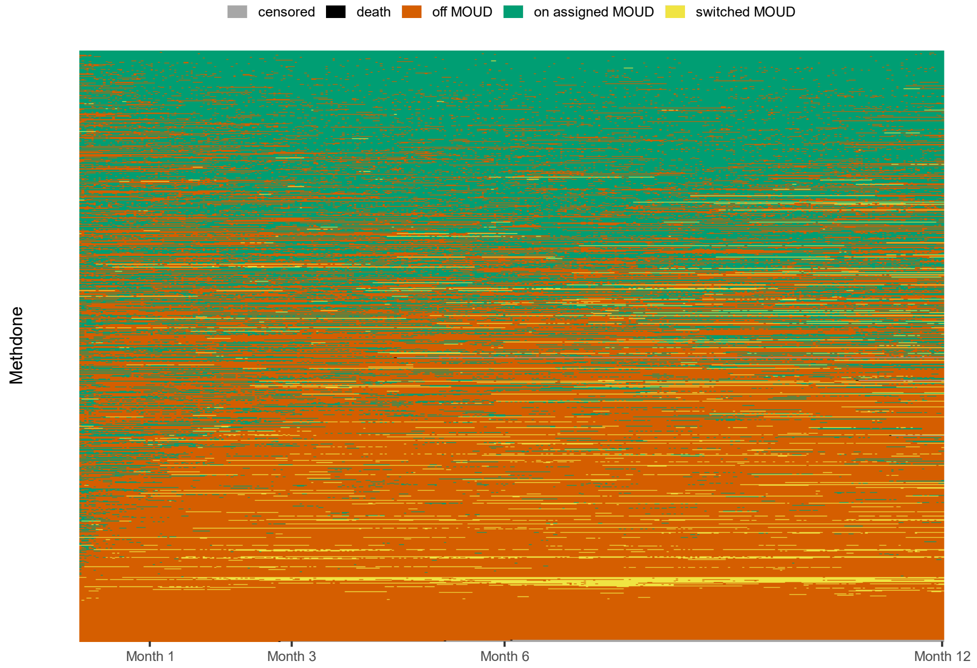

© 2026 Socias ME et al. *JAMA Network Open*.

eReferences

1. Nosyk B, Min JE, Homayra F, et al. Buprenorphine/Naloxone vs Methadone for the Treatment of Opioid Use Disorder. *JAMA*. 2024;332(21):1822-1831. doi:10.1001/jama.2024.16954

2. Janjua NZ, Islam N, Kuo M, et al. Identifying injection drug use and estimating population size of people who inject drugs using healthcare administrative datasets. *Int J Drug Policy*. 2018;55:31-39. doi:10.1016/j.drugpo.2018.02.001

3. BC Ministry of Health – Health Sector Information, Analysis and Reporting (HSIAR) Division. B.C. Health System Strategy. Geographic Service Areas. Published online March 7, 2023.

4. Quan H, Sundararajan V, Halfon P, et al. Coding algorithms for defining comorbidities in ICD-9-CM and ICD-10 administrative data. *Med Care*. 2005;43(11):1130-1139. doi:10.1097/01.mlr.0000182534.19832.83

5. Larochelle MR, Lodi S, Yan S, Clothier BA, Goldsmith ES, Bohnert ASB. Comparative Effectiveness of Opioid Tapering or Abrupt Discontinuation vs No Dosage Change for Opioid Overdose or Suicide for Patients Receiving Stable Long-term Opioid Therapy. *JAMA Netw Open*. 2022;5(8):e2226523. doi:10.1001/jamanetworkopen.2022.26523

© 2026 Socias ME et al. *JAMA Network Open*.
