# Supplementary material for: Slow-Release Oral Morphine vs Methadone for Opioid Use Disorder in the Fentanyl Era
Source: JAMA Netw Open. 2026 Mar 24;9(3):e262970. doi: 10.1001/jamanetworkopen.2026.2970 (PMC13014207; doi:10.1001/jamanetworkopen.2026.2970)
Supplement: Supplement 2. — Data Sharing Statement [file jamanetwopen-e262970-s002.pdf]

# Data Sharing Statement

Socias. Slow-Release Oral Morphine vs Methadone for Opioid Use Disorder in the Fentanyl Era. *JAMA Netw Open*. Published March 24, 2026. doi:10.1001/jamanetworkopen.2026.2970

**Data**
**Data available:** No

**Additional Information**
**Explanation for why data not available:** The datasets generated and/or analyzed during the current study are not publicly available due to concerns on individual level administrative data and privacy restrictions. Materials and code available on request.
